# Surface state-induced barrierless carrier injection in quantum dot electroluminescent devices

**DOI:** 10.1038/s41467-021-25955-z

**Published:** 2021-09-27

**Authors:** Hyeonjun Lee, Byeong Guk Jeong, Wan Ki Bae, Doh C. Lee, Jaehoon Lim

**Affiliations:** 1 Department of Chemical and Biomolecular Engineering, KAIST Institute for the Nanocentury, Korea Advanced Institute of Science and Technology (KAIST), Daejeon, 34141 Republic of Korea; 2 SKKU Advanced Institute of Nanotechnology (SAINT), Sungkyunkwan University (SKKU), Suwon, Gyeonggi-do 16419 Republic of Korea; 3 Department of Energy Science, Centre for Artificial Atoms, Sungkyunkwan University (SKKU), Suwon, Gyeonggi-do 16419 Republic of Korea

**Keywords:** Electronic devices, Quantum dots, Quantum dots

## Abstract

The past decade has witnessed remarkable progress in the device efficiency of quantum dot light-emitting diodes based on the framework of organic-inorganic hybrid device structure. The striking improvement notwithstanding, the following conundrum remains underexplored: state-of-the-art devices with seemingly unfavorable energy landscape exhibit barrierless hole injection initiated even at sub-band gap voltages. Here, we unravel that the cause of barrierless hole injection stems from the Fermi level alignment derived by the surface states. The reorganized energy landscape provides macroscopic electrostatic potential gain to promote hole injection to quantum dots. The energy level alignment surpasses the Coulombic attraction induced by a charge employed in quantum dots which adjust the local carrier injection barrier of opposite charges by a relatively small margin. Our finding elucidates how quantum dots accommodate barrierless carrier injection and paves the way to a generalized design principle for efficient electroluminescent devices employing nanocrystal emitters.

## Abstract (web summary)

Hybrid quantum dot light-emitting diodes exhibit barrier-less carrier injection despite a seemingly unfavourable energy landscape. Here, Lee et al. unravel the origin of this barrier-less carrier injection, showing the critical role of surface states of quantum dots.

## Introduction

The impetus for display devices with high color purity and low power consumption has driven intensive research on quantum dot-based light-emitting diodes (QLEDs) to exploit narrow bandwidth and high emission quantum yield (QY) of quantum dots (QDs). The QLED performance has improved hand in hand with the advances in the wet-chemical synthesis of colloidal QDs and device architectures^1–3^. In particular, the key to the progress in QLED efficiency is the use of organic–inorganic hybrid device architecture (denoted as hybrid QLEDs hereafter)^4^. It turned out that some combinations of metal oxide (e.g., ZnO) electron transport layers (ETLs) and organic hole transport layers (HTLs) effectively mitigate the issue of uneven carrier injection into emissive QD layer^5,6^. As opposed to organic ETLs with small electron affinity (2.0–3.0 eV)^7^, ZnO ETLs exhibit considerably large electron affinity (~4.0 eV). This contributes to balancing the electron and hole injection rates that would be impeded by a large gap between ionization energy of QD core (e.g., CdSe, CdS, or InP) and typical organic HTLs^8,9^.

**Fig. 1:**
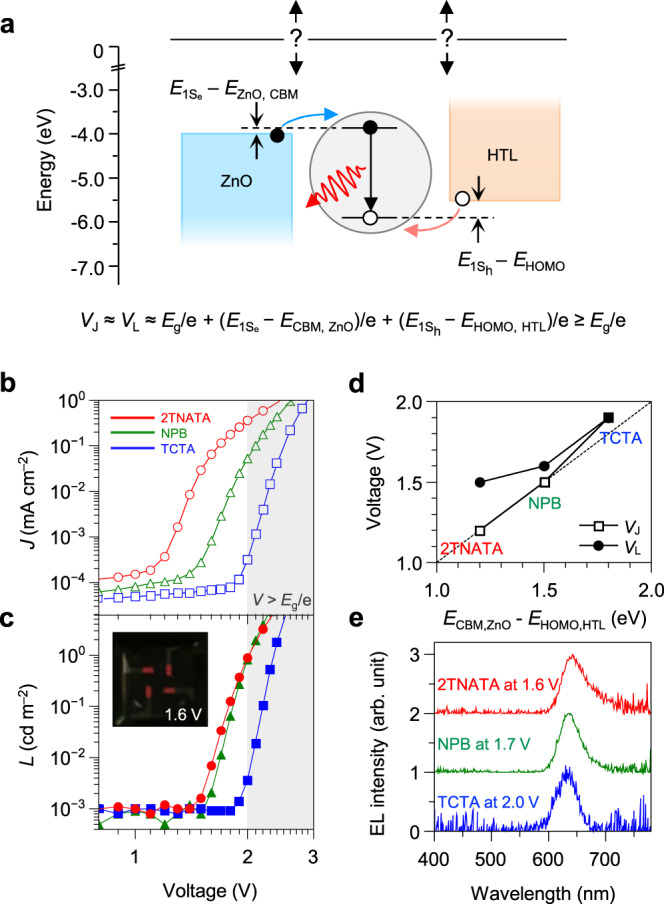
Sub-band gap operation of quantum dot light-emitting diodes with energy band discontinuity. **a** Energy level diagram for materials used in this study without taking the interface modification into consideration. The equation estimates the current threshold (*V*_J_) of QLEDs under the assumption that series resistance is negligible. **b** Current density (*J*)–voltage (*V*) (open symbols) and **c** luminance (*L*)–voltage (closed symbols) characteristics of QLEDs employing 2TNATA (red circle), NPB (green triangle), and TCTA (blue square) HTLs. Grey shaded region indicates *V* > *E*_g_ /e (=1.97 V), where *E*_g_ is a bandgap of QDs. Inset shows a photographic image of 2TNATA-based QLEDs taken at an applied bias of 1.6 V. The pixel size of devices is 1.4 × 3.0 mm^2^. **d** *V*_J_ and luminance thresholds (*V*_L_) plotted to *E*_CBM, ZnO_ − *E*_HOMO, HTL_, where *E*_CBM, ZnO_ is the CBM of ZnO and *E*_HOMO, HTL_ is a HOMO level of HTL. Dashed line indicates *V* = (*E*_CBM, ZnO_ − *E*_HOMO, HTL_)/e for eye guidance. **e** EL spectra of QLEDs with different HTLs acquired at near *V*_L_.

In hybrid QLEDs, an interesting yet under-investigated question remains to be answered. There are considerable energy level differences between the conduction band minimum (CBM) (*E*_CBM_) of ZnO and the electron state of emissive core ($E_{{{\mathrm{1S}}}_{e}}$) and between the hole state of emissive core ($E_{{{\mathrm{1S}}}_{h}}$) and the highest occupied molecular orbital (HOMO) level of the HTL (*E*_HOMO, HTL_). This means that one needs to apply external bias exceeding the optical band gap (*E*_g_) of QDs to obtain EL from QLEDs. *E*_g_, $E_{{{\mathrm{1S}}}_{e}}$ − *E*_CBM, ZnO_, and $E_{{{\mathrm{1S}}}_{h}}$ − *E*_HOMO, HTL_ are supposed to add up to the turn-on voltage of QLEDs (Fig. 1a)^10^. Interestingly, most hybrid QLEDs exhibit current density and light emission threshold voltages (*V*_J_ and *V*_L_, respectively) comparable to or lower than *E*_g_/e despite large $E_{{{\mathrm{1S}}}_{h}}$ − *E*_HOMO, HTL_ (Supplemental Table 1). The thermal effect, which is in the scale of up to ~4*k*T/e or ~0.1 V, can hardly account for the sub-*E*_g_ turn-on^10^. It appears that ZnO “electron” transport materials in the hybrid QLEDs trigger barrierless “hole” injection.

Explanations for such barrierless carrier injection observed exclusively in hybrid QLEDs could include Auger-assisted up-conversion^11^ or thermionic emission^12^. The former proposes excitation of the hole by excess energy of charge transfer exciton recombining at the ETL–HTL or the QD–HTL interface. However, a large separation between ETLs and HTLs by a 10–20 nm-thick QD emissive layer (EML) makes the generation of charge-transfer excitons less probable. Recent QLEDs with sub-*E*_g_ turn-on employing QDs with large electron confinement^13–16^ make it difficult to generalize this hypothesis proposed for CdSe/CdS QDs with negligible electron confinement^5^. Moreover, the general behavior of recent hybrid QLEDs, increasing internal quantum efficiency beyond threshold voltage and even reached to near unity, would not be validated. If it involves since the device turn-on, intensifying the Auger process proportional to *n*^3^ (*n*: carrier concentration) would strongly degrade internal quantum efficiency with increasing current^17^. On the other hand, the thermionic emission process involves the possibility of the sub-*E*_g_ emission based on a correlation between a number of thermally excited holes and current density. However, this proposed mechanism cannot provide a sufficient answer as to why only ZnO-based ETLs enable the sub-*E*_g_ operation of QLEDs while organic ETLs tend to exhibit thresholds larger than a flat band bias (Supplementary Table 1).

In addition to the limitations of these models, there is another caveat that profoundly concerns the validity of the mechanisms. In the line of reasoning, QDs and charge transport layers (CTLs) are treated as dielectrics for the simplicity of argument, perhaps to a fault. This excessive simplification is basically a result of assimilation of organic light-emitting diodes, in which work functions (*ɸ*) of organic CTLs similarly locate at the middle of *E*_g_ and few carriers (~10^10^ cm^−3^ or less)^18^ are exchangeable at the junction. Core/shell QDs are almost insulating and confine carriers with wide-*E*_g_ shells, thus seemingly unable to provide carriers to be exchanged at the interface. To make sense of the barrierless carrier injection in hybrid QLEDs, however, it is necessary to consider the overall energy landscape of QLEDs including energy level equilibrium between QDs and CTLs.

In this work, we elucidate that the barrierless hole injection in hybrid QLEDs originates from the electrostatic potential gain at the QD–HTL interface. Macroscopic energy landscape is reorganized along the Fermi level of QDs derived by their surface states and this modified interfacial structure allows holes to be injected into QDs. Meanwhile, the discrete nature of individual QDs with different *E*_g_ spawns the difference in electron occupation of QDs. While the Coulombic potential of an electron in QDs to the hole is marginal compared to the extent of macroscopic potential gain, at sub-*E*_g_ condition, it modulates the exciton generation rate of individual QDs against nonradiative carrier loss through surface states. Poor device performance at sub-*E*_g_ condition can be translated into the dominance of trap-involved nonradiative carrier loss through neutral wide-*E*_g_ QDs that is incapable of promoting charge injection by Coulombic attraction. We believe that our findings clarify how QDs accommodate barrierless carrier injection in hybrid QLEDs and pave the way to a generalized design principle for efficient electroluminescent devices employing nanocrystal (NC) emitters.

## Results and discussion

For our study, we adopt the inverted device structure employing a ZnO nanoparticle (NP) ETL, InP/ZnSe/ZnS core/multishell QDs (Supplementary Fig. 1) and various organic HTLs with different ionization energies for systematic investigation: 4,4′,4″-tris[2-naphthyl(phenyl)amino]triphenylamine (2TNATA, 5.1 eV), N,N′-di(1-naphthyl)-N,N′-diphenyl-(1,1′-biphenyl)-4,4′-diamine (NPB, 5.4 eV), and tris(4-carbazoyl-9-ylphenyl)amine (TCTA, 5.7 eV). In current density (*J*)–voltage (*V*)–luminance (*L*) characteristics of QLEDs, we observe an intriguing and somewhat counterintuitive trend of *V*_J_ and *V*_L_ upon using various HTLs. In the case of HTL with a larger $E_{{{\mathrm{1S}}}_{h}}$ − *E*_HOMO, HTL_ value, both *V*_J_ and *V*_L_ are lower than what would correspond to the bandgap of QDs (*E*_g_/e = 1.97 eV) (Fig. 1b, c). For example, *V*_J_ = 1.2 V and *V*_L_ = 1.5 V when 2TNATA was used as a HTL. One would attribute the sub-*E*_g_ thresholds to the recombination of charge transfer complex because *V*_J_ coincides with built-in potential, *E*_CBM, ZnO_ − E_HOMO, HTLs_ (Fig. 1d). However, EL spectra acquired in the range of *V*_L_ ≤ *V* ≤ *E*_g_/e suggest that the carriers are injected into QDs at this regime, as opposed to recombining in CTLs or via charge-transfer excitons (Fig. 1e).

**Fig. 2:**
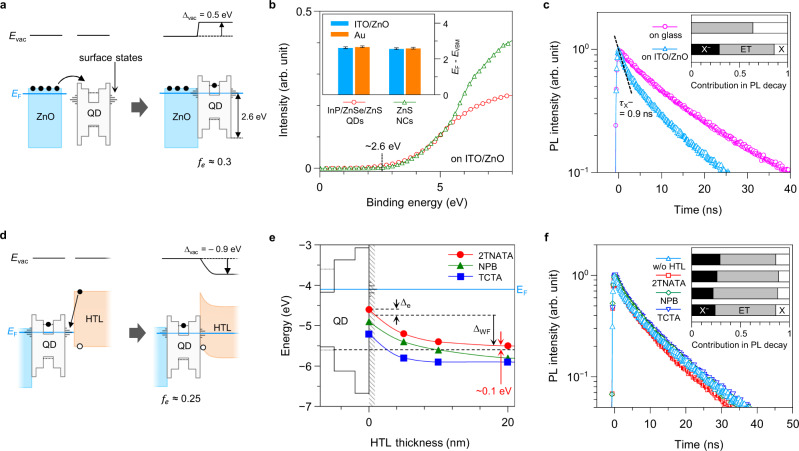
Interfacial electrostatic potential gain by surface state-derived Fermi level alignment. **a** Modification of energy levels at a ZnO ETL–QD EML junction. Blue solid line represents Fermi level (*E*_F_). Vacuum level shift (Δ_vac_) is estimated to be 0.5 eV. **b** UPS spectra of InP/ZnSe/ZnS QDs (red square) and ZnS NCs (green triangle) on ITO/ZnO substrates at low binding energy region. Inset shows the *E*_F_ − *E*_VBM_ values on ITO/ZnO (blue) and Au (orange) substrates. Error bars indicate an instrument resolution, 0.05 eV. **c** PL decay dynamics of QDs on glass (pink circle) and ITO/ZnO substrates (sky blue triangle). X^−^ decay occurs at an early stage in the case of ITO/ZnO, with a negative trion lifetime ${\tau}_{{{{\mathrm{X}}}}^{-}}$ = 0.9 ns. Inset displays fractions of decay channels: X^−^ (black), neutral exciton (X, white), and delayed recombination by energy transfer (ET, grey). **d** Band bending at the QD–HTL junction with Δ_vac_ = −0.9 eV. **e** HOMO levels of 2TNATA (red circle), NPB (green triangle), and TCTA (blue square) on ITO/ZnO/QDs substrates as a function of HTL thickness. **f** PL decays of QDs in ITO/ZnO/QDs/HTLs: without HTLs (sky blue triangle), 2TNATA (red square), NPB (green diamond), and TCTA (blue inverse triangle). Inset shows the contribution of X^−^ (black), X (white), and ET (grey). All PL decays were acquired at the entire emission range with excitation at 488 nm.

To analyse the mechanism of the barrierless injection at interfaces, we probe the energy level of each component in QLEDs using ultraviolet photoelectron spectroscopy (UPS). Interestingly, InP/ZnSe/ZnS QDs on ZnO (ITO/ZnO/QDs) exhibit the Fermi level (*E*_F_) at ~2.6 eV higher than the apparent VBM of QDs (*E*_F_ − *E*_VBM, QD_ = 2.6 eV; Fig. 2a, b and Supplementary Fig. 2). This value is identical when Au electrode was used in lieu of the ITO/ZnO substrate (Inset in Fig. 2b), suggesting the *E*_F_ pinning of QD films. To elucidate whether the *E*_F_ pinning occurs by cores or shells of the QDs, we perform a comparative study using ZnS NCs with similar size and surface chemistry (Supplementary Figs. 3 and 4). The *E*_F_ − *E*_VBM, ZnS_ values of ZnS NCs on ITO/ZnO and Au substrates are both ~2.6 eV (Fig. 2b and Supplementary Fig. 4). This value corresponds to the surface state emission of ZnS NCs centered at 2.6 eV (Supplementary Fig. 3a) which is associated with undercoordinated Zn atoms on the surface^19,20^ leaving electron-accepting states closed to CBM^21^ (Inset in Supplementary Fig. 3a), not bearing on 1S_e_ state of the InP core (dotted lines in Fig. 2a; see Supplementary Note 1 for details). Clear correspondence of *E*_F_ − *E*_VBM_ between QDs and ZnS NCs excludes the involvement of In-, P-, or Se-related defect states inside QDs. Our observation clarifies that *E*_F_ and energy levels examined by UPS solely reflect characteristics of the outmost ZnS shell and it determines the relative energy level position of QDs to ZnO.

The ZnO–QD junction formation seems to accompany charge transfer to equilibrate *E*_F_. Due to a high electron concentration (~10^16^ cm^−^^3^)^22^ and lower *ɸ* of ZnO ETL compared to QDs, electrons in ZnO ETL tend to migrate to adjacent electronic states of QDs, such as surface states and/or 1S_e_ states. The *ɸ* difference between the ZnO ETL and the QDs without a junction (Δ_*ɸ*, ZnO–QD_ = 0.35 eV; see Supplementary Fig. 2e) shows a disparity with observed Δ_vac, ZnO–QD_ of 0.5 eV. We attribute the offset Δ_vac, ZnO–QD_ − Δ_*ɸ*, ZnO–QD_ to the spontaneous electron charging to 1S_e_ states in QD EMLs. Based on the comparative study of QDs on glass, transient PL decay of ITO/ZnO/QDs taken at the entire emission range gives rise to the negative trion (X^–^) decay with a fraction (*f*_e_) of ca. 30% and the X^–^ lifetime ($\tau_{{{{\mathrm{X}}}}^{-}}$) of 0.9 ns (Fig. 2c and Supplementary Table 2) that coincides with the X^–^ decay lifetime extracted from photodoped QDs^23^ (see Methods and Supplementary Fig. 5). A modified Helmholtz equation^24^ yields the electrostatic potential of the partially-charged QD EML (∆_e_) to be ∆_e_ = 0.18 eV for *f*_e_ = 0.3, in close proximity with Δ_vac, ZnO–QD_ − Δ_*ɸ*, ZnO–QD_ = 0.15 eV (Supplementary Note 2). Overall, the energy level alignment at the ZnO–QD junction is affected not only by the *ɸ* disparity but also by the electrons injected into the QD core.

Interestingly, the surface state-derived *E*_F_ of QDs casts a profound impact on the energy landscape at the QD–HTL interface. UPS analysis on the thickness-dependent energy level of HTLs on ITO/ZnO/QDs (ITO/ZnO/QDs/HTLs) reveals that gradual band bending of 0.8–0.9 eV develops up to ~10 nm from the junction in the case of all tested HTLs (Supplementary Fig. 6 and Fig. 2d, e). The extent of the band bending corresponds to the *ɸ* difference between the QDs and the HTLs without junction (Δ_*ɸ*, QD–HTL_ = 0.65−0.75 eV; see Supplementary Fig. 6) on top of the contribution of negatively charged QD EMLs after deposition of HTLs (∆_e_ = 0.13–0.15 eV), estimated from PL decay dynamics of ITO/ZnO/QDs/HTLs (Fig. 2f and Supplementary Table 2). We attribute the origin of band modification to migration of thermally- or optically-generated (e.g., excitation by ambient light) electrons in HTLs to QDs; electrons are transferred to the surface states on QDs, and the remaining holes accumulate at the QD–HTL interface as space charge. We observed a minimal reduction of *f*_e_ around 3–7% point (Fig. 2f and Supplementary Table 2) using QD-selective low energy excitation at 488 nm (see Methods for details). The decrease in *f*_e_ attests to the recombination between electrons in 1S_e_ states and holes at the QD–HTL junction at equilibrium, partially allowed by the electron delocalization to surface states and a large number of holes at the interface. Summing up, the electrons seem to migrate not to the 1S_e_ states, but to the surface states on QDs during equilibrium.

The series of observations offer a testament that the energy level landscape in hybrid QLEDs is explained more clearly when QDs are treated as semiconductors, not as dielectrics. Building on the premise, we can make better sense of the sub-*E*_g_ turn-on in the hybrid QLEDs. First, the band bending at the QD–organic HTL junction reduces the energy level offset for the hole injection, $E_{{{\mathrm{1S}}}_{h}}$ − E_HOMO, HTL_, in the way of large electrostatic potential gain. This gain effectively lowers the hole injection barrier: e.g., for 2TNATA from 0.9 eV (non-aligned) to ~0 eV, as manifested from ~0.1 eV from UPS analysis (red arrows in Fig. 2e). Second, the electron injection barrier, $E_{{{\mathrm{1S}}}_{e}}$ − *E*_CBM, ZnO_, is raised as a result of the *E*_F_ alignment, yet the increase is marginal since *E*_F_ difference is merely 0.35 eV. That is attributed to that both the ZnO ETL and the Zn chalcogenide shell have defect states derived by undercoordinated Zn atoms on their surface^25,26^. In addition, the high electron concentration of ZnO narrows the electron injection barrier width^27^. In the hybrid QLEDs examined in this work, overall electrostatic potential gain at the ZnO–QD–2TNATA interfaces (i.e., Δ_vac, ZnO–QD_ + Δ_vac, QD–HTL_) effectively lowers the total energy barrier for carrier injection into QDs by ~0.4 eV, which agrees with the offset between *V*_L, 2TNATA_ and *E*_g_/e of QDs. As most QDs studied in previous reports feature the zinc chalcogenide shell, the theory of band bending and *E*_F_ pinning could offer hindsight for the sub-*E*_g_ operation in hybrid QLEDs.

The explanation extends to the limitation of all-organic QLEDs with *V*_L_ > *E*_g_/e. At the QD–organic ETL junctions, one would estimate the energy barrier for electron injection to be large due to the *ɸ* difference: for instance, ~0.45 eV for 3,5-tri(phenyl-2-benzimi-dazolyl)-benzene (TPBi)^28^ or ~0.75 eV for tris-(8-hydroxyquinoline)aluminium (Alq_3_)^29^. The organic ETLs are likely to impose a large and wide electron injection barrier at the QD–organic ETL junction due to low carrier concentration (~10^10^ cm^−^^3^ or less)^18^, consequently resulting in increased operation thresholds (Supplementary Table 1).

**Fig. 3:**
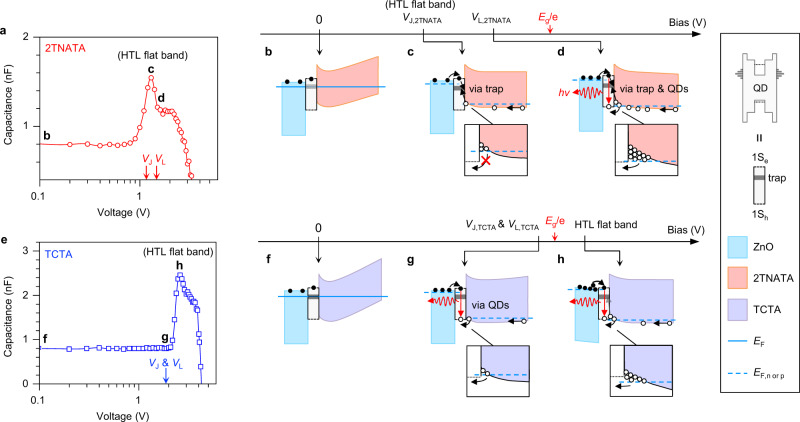
Carrier accumulation and injection under modified energy band landscape. **a** Capacitance (*C*)–voltage (*V*) characteristic of 2TNATA-based QLEDs. Current (*V*_J_) and luminance thresholds (*V*_L_) are 1.2 eV and 1.5 eV, respectively (red arrows). **b**–**d** Band diagrams of ZnO/QD/2TNATA with increasing bias, from *V* = 0 V (**b**), to 1.2 V (**c**), and to 1.5 V (**d**). **e** A *C*–*V* characteristic of TCTA-based QLEDs. *V*_J_ and *V*_L_ are identical to 1.9 eV (blue arrow). **f**–**h** Band diagrams of ZnO/QD/TCTA with increasing bias, from *V* = 0 V (**f**), to 1.9 V (**g**), to 2.6 V (**h**). Legend for **b**–**d** and **f**–**h** is shown on the right panel. Blue lines represent the *E*_F_ at equilibrium (solid line) and the quasi-Fermi level of electron (*E*_F,n_) and hole (*E*_F,p_) under applied bias (dashed line). We note that the quasi-*E*_F_ of HTLs is defined by molybdenum oxide/Al anode, assign at ~0.3 eV smaller than *E*_HOMO, HTL_.

Despite the closeness of electrostatic potential gain from HTLs, *V*_L_ is larger than *V*_J_ in the hybrid QLEDs with 2TNATA and NPB, while *V*_L_ and *V*_J_ are in similarly close proximity to *E*_g_/e in TCTA-based devices (Fig. 1d). To rationalize such observation, we perform the capacitance (*C*)–voltage (*V*) analysis for 2TNATA (Fig. 3a–d) and TCTA-based QLEDs (Fig. 3e–h). We note that the increase in capacitance and energy band deformation up to *V* ≈ *V*_J_ occurs mainly in organic HTLs. The ZnO ETL remains flat nearly up to the threshold voltage^30^ due likely to its high dielectric constant (relative dielectric constant: ~8)^31^ and carrier concentration (~10^16^ cm^−3^)^22^. The applied bias of 1.1 V for 2TNATA and 2.6 V for TCTA at peak capacitances represent a flat band condition of HTLs (Fig. 3c, h for respective HTL)^32^.

We found that the thresholds of 2TNATA-based QLEDs take place as holes accumulate at the QD–HTL interface. Based on the following experimental findings, we attribute the threshold behavior of 2TNATA-based QLEDs to the influence of surface defects (Fig. 3c). First, a hybrid QLED employing ZnS NCs exhibits a trap-filled limit voltage (*V*_TFL_) at 1.0 V (Supplementary Fig. 7a). Second, a *L*–*J* characteristic around the *V*_L, 2TNATA_ shows a super-linear behavior with a slope of ~2.3 (Supplementary Fig. 7b), suggesting the involvement of the surface states^33^, not the Auger recombination of carriers (*L ∝ J*^2/3^)^34^ and corresponding energy up-conversion^11,35^. Thus, trap-involved recombination is initiated at *V* = *V*_J, 2TNATA_ and competes with the hole injection into QDs despite at *V* ≥ *V*_L, 2TNATA_ (Fig. 3d). While the potential gain at the QD–2TNATA of 0.9 eV allows for hole injection at *V* ≥ *V*_L, 2TNATA_, the leakage current exceeds the exciton recombination current by *V* = ~3 V (Supplementary Fig. 7b). We rule out multiexcitons in this regime since *J* is too low compared to exciton recombination rate^36^.

On the other hand, for both *V*_J, TCTA_ and *V*_L, TCTA_ of TCTA-based QLEDs are ~1.9 V, consistent with the onset of capacitance increase (Fig. 3e). While the potential does not lead to a flat band of TCTA, the bias exceeding the built-in potential of given devices (*E*_CBM, ZnO_ − *E*_HOMO, HTLs_ = 1.8 eV) would result in the migration of thermally generated holes to the 1S_h_ state without considerable hole accumulation at the interface (Fig. 3e, g). Almost linear *L–J* relationship above *J* = 10^−2^ mA cm^−2^ (Supplementary Fig. 7b) and *V*_TFL_ of a device with ZnS QDs as large as 2.3 V (Supplementary Fig. 7a) suggest that the carrier leakage via traps has little to do with *J*, and hence nearly identical *V*_J, TCTA_ and *V*_L, TCTA_. While holes accumulate at the junction at increased *V*, the accelerated hole injection driven by sufficient electrostatic potential (dashed lines in Fig. 3h, $E_{{{\mathrm{1S}}}_{h}}$ − E_HOMO, TCTA_ = −0.3 eV) lowers the concentration of holes at the interface and reduces trap-assisted leakage.

The intervention of the trap states in carrier injection to QDs necessarily results in degradation of device performance because it lowers charge injection efficiency (*η*_inj_) in the equation of external quantum efficiency (EQE): *η*_EQE_ = *η*_QY, EML_ × *η*_inj_ × *η*_out_, where *η*_QY, EML_ is a PL QY of QD EMLs, *η*_inj_ is a carrier injection efficiency, and *η*_out_ is an outcoupling efficiency. Assuming that only neutral QDs are emissive [*η*_QY, EML_ = *η*_QY, film_ × (1 − *f*_e_), where *η*_QY, film_ = 0.41 and *f*_e_ = ~0.3 at peak EQE condition; see Supplementary Fig. 8a, b] and an outcoupling efficiency is ~0.2^8^, the theoretical maximum EQE of QLEDs is ~5.7% when *η*_inj_ = 1. An EQE curve of TCTA-based QLEDs ranging at ~5% in Supplementary Fig. 8c concludes the suppressed carrier loss via trap states and corresponding high *η*_inj_ = ~0.88. In the case of 2TNATA-based QLEDs, on the contrary, the trap-related carrier loss is initiated even before the device turn-on, consequently, the EQE becomes as low as ~2% due to poor *η*_inj_ = ~0.35.

Macroscopic investigation on the energy landscape and charge accumulation in this study provides a clear understanding on the energetics of carriers in hybrid QLEDs, how the carrier injection barrier can be relaxed by the electrostatic potential gain at the junctions. In fact, this macroscopic postulation has paved the way for various approaches promoting balanced carrier injection to QD EMLs, for instance, controlling confinement barriers^8,37^ by the shell that is demonstrated in typical Cd-based QLEDs. The line of logic is based on the notion that the QD EMLs have continuum emissive states in which each state is commutable and carrier injection rates reflect average carrier occupation of entire emissive states, as is the case in conventional inorganic and organic EL devices. However, in the case of QD EMLs, the wide-*E*_g_ shell and surface ligands isolate the energy states of cores, which impede the migration of charges in the planar direction. A complication of carrier injection processes arisen from the discrete nature of each QD is a main subject of the following discussion.

**Fig. 4:**
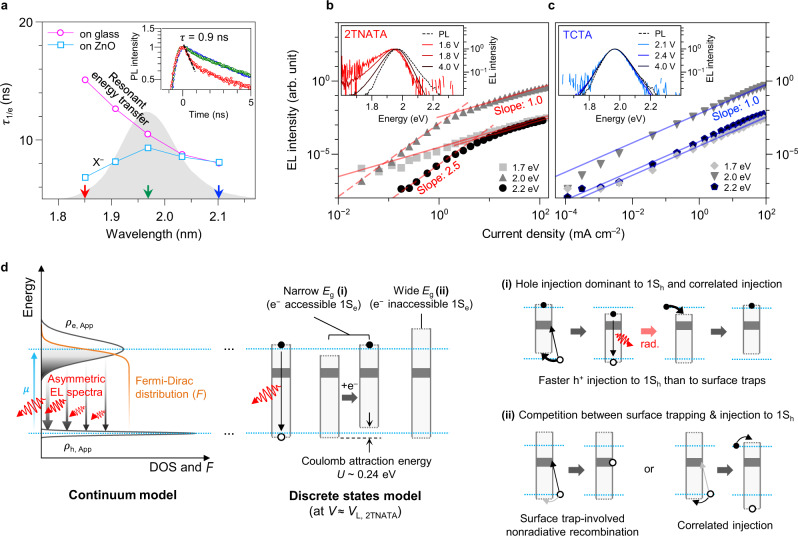
Local electrostatic potential gain granted by a charge in individual QDs and discrete nature of QD emissive layer. **a** Spectrally resolved PL decay lifetime (*τ*_1/e_) of glass/QDs (magenta circle) and ITO/ZnO/QD (sky blue square) at different wavelengths. PL spectrum of QD film is provided as grey background. Inset shows PL decay traces of ITO/ZnO/QDs at 2.10, 1.97, and 1.85 eV, indicated using blue, green, and red arrows, respectively, on the *x*-axis of the main panel. X^–^ decay is clearly observed at 1.85 eV with a lifetime of 0.9 ns in early stage (red). **b**, **c** EL intensity–*J* dependence probed at 1.7 eV (light grey square), 2.0 eV (dark grey triangle), and 2.2 eV (black circle) for 2TNATA-based (**b**) and TCTA-based QLEDs (**c**). Dashed and solid lines indicate regression lines with a slope of 2.5 and 1.0, respectively. Insets show EL spectra acquired at different biases. **d** Schematic illustration on continuum density of state (DOS) model. At a specific electron chemical potential (*μ*, sky blue line), electrons occupy the apparent 1S_e_ DOS (*ρ*_e, App_) from the lowest state following the Fermi–Dirac distribution (*F*), *F* × *ρ*_e, App_. On the other hand, holes in the apparent 1S_h_ DOS (*ρ*_h, App_) possess minimal energy distribution due to the narrowness of *ρ*_h, App_. Spectral shape is therefore determined by the distribution of *F* × *ρ*_e, App_ in energy scale that shows low-energy tail. **e** In the microscopic viewpoint, individual QDs experience different electron occupation, and carrier injection events. In 2TNATA-based QLEDs, at *V* ≈ *V*_L_, prior injection of an electron into narrow-*E*_g_ QDs results in prompt injection of a hole via Coulombic attraction (*U*) [(i), upper right]. In the case of wide-*E*_g_ QDs, a hole is likely to migrate to 1S_h_ state or to surface states. Barring the unlikely event that the hole introduced to 1S_h_ state electrostatically attracts an electron, the trap-involved nonradiative recombination is dominant.

The electron charging of QDs on ITO/ZnO substrates in QLEDs turns out to depend on *E*_g_ of QDs, which highlights the effect of variation of individual QDs in the EML. The opposite tendency observed in spectrally-resolved PL decay^38^ of narrow-*E*_g_ QDs on ITO/ZnO and glass substrates suggests selective electron transfer to narrower-*E*_g_ QDs thanks to lower electron injection barrier (Fig. 4a). For example, ITO/ZnO/QDs develops a fast decay pathway with a lifetime of 0.9 ns at 1.85 eV (A red arrow in Inset of Fig. 4a and Supplementary Fig. 9) corresponding to $\tau_{{{{\mathrm{X}}}}^{-}}$ (Supplementary Fig. 5) while the glass/QDs exhibit a longer lifetime due to resonant energy transfer (ET). The *E*_g_ variation of QDs makes a marginal impact on $E_{{{\mathrm{1S}}}_{h}}$ because of the considerably heavier effective mass of a hole (*m*_h_*) than that of an electron (*m*_e_*) in InP (7.5*m*_e_* ≈ *m*_h_*; see Supplementary Fig. 2e)^39^. The variation of 1S_h_ state between the narrowest and widest-*E*_g_ QDs is merely up to ~41 meV for a given QD ensemble.

Under applied bias *V* ≈ *V*_L_, we indeed observe asymmetric and red-shifted emission profiles from 2TNATA-based QLEDs, seemingly reflecting dominant participation of narrow-*E*_g_ QDs in EL (Inset in Fig. 4b). However, the EL spectra from TCTA-based QLEDs appear unaltered from the PL spectra of QD EML (inset in Fig. 4c and Supplementary Fig. 10a). To make sense of the skewed EL spectra in the case of the 2TNATA-based device, we introduce the continuum density of states (DOS) model that translates the EL spectrum as recombination of electrons in the broad 1S_e_ DOS with holes in the narrow 1S_h_ DOS with identical electron and hole injection rates (Fig. 4d; see Supplementary Note 3). As shown in Supplementary Fig. 10b, the theoretical results corroborate the EL spectrum of 2TNATA devices at *V* ≈ *V*_L_ with an offset of overall potential gain. However, this model fails to account for the *V*-dependent EL peak energies and spectral shapes in the case of 2TNATA-based devices. According to the model, *V* ≥ 2 V is sufficient to inject electrons to wide-*E*_g_ QDs, contradicting the observed asymmetric and red-shifted EL. The experimental results speak volumes for the effect of individual characteristics of QDs; the use of specific HTLs gives rise to the substantial differences between QDs in carrier injection rates, charge occupations, and exciton generation rates.

The spectrally resolved EL intensity from QLEDs recorded at various *J* offers a deeper understanding of the mechanism of the carrier injection into QDs. For 2TNATA- and TCTA-based devices, we investigate the EL intensity–*J* relationship for narrow-, intermediate-, and wide-*E*_g_ QDs with emission energies *E*_emission_ = 1.7, 2.0, and 2.2 eV, respectively (Fig. 4b, c). The EL intensity in the 2TNATA-based QLEDs exhibits a transition from super-linear (slope: ~2.5) to linear *J*-dependence for wide-*E*_g_ QDs (*E*_emission_ = 2.0 and 2.2 eV), while a linear correlation persists in the measured current density range for narrow-*E*_g_ QDs (*E*_emission_ = 1.7 eV) (Fig. 4b). Interestingly, in the case of the TCTA-based devices, no such a transition is observed and the linear dependence prevails regardless of *E*_g_ of QDs (Fig. 4c). Now that the super-linear relationship is associated with the intervention of the trap-involved nonradiative pathway (Supplementary Fig. 7b), varied slopes of the EL intensity–*J* for different *E*_g_ result from the varying carrier injection rate into different QDs. In other words, narrow-*E*_g_ QDs primarily consume holes via exciton recombination although a larger number of holes at the QD–2TNATA interface may cause trap recombination for all QDs. This bias dependence is rather counter-intuitive in a model where the macroscopic electrostatic potential allows for initiating hole injection into 1S_h_ states of all QDs owing to the minimal energy difference in 1S_h_. And Auger-assisted carrier injection mechanism^5,35^ cannot rationalize our observation because the up-conversion energy (*E*_CBM, ZnO_ − *E*_HOMO, HTLs_, 1.2–1.8 eV)^40^ exceeds the *E*_g_ difference of QDs. Therefore, the *E*_g_-dependent EL intensity demands an explanation as to why hole injection is facilitated in narrow-*E*_g_ QDs.

In the process of correlated charge injection^36^, an electron is injected into emissive materials prior to hole injection, which provides local Coulombic attraction (*U*) and consequent enhancement of hole injection rate. To fully deconstruct the carrier injection processes in this context, we made further analysis on the 2TNATA-based devices. At *V* < *V*_L_, narrow-*E*_g_ QDs are charged with an electron and wide-*E*_g_ QDs are neutral [(i) and (ii) in Fig. 4e, respectively]. The electron in a narrow-*E*_g_ QD alters the 1S_h_ state by *U* = −0.24 eV for given core size^41^ (Fig. 4e and Supplementary Note 4), which facilitates the hole injection by lowering $E_{{{\mathrm{1S}}}_{h}}$ − E_VBM, HTLs_ [(i) in Fig. 4e]. At bias *V* ≥ *V*_L_, this local potential gain promotes a formation of neutral exciton and results in the linearity in EL intensity–*J* curve (Fig. 4b). On the other hand, the large electron injection barrier (~0.5 eV) in the neutral wide-*E*_g_ QDs barely allows electrons from ZnO into the 1S_e_ states at *V* ≈ *V*_L_. Without Coulombic assistance, the hole injection in 1S_h_ states would compete with the trap-involved process [(ii) in Fig. 4e]. The super-linear regime of wide-*E*_g_ QDs in the EL intensity–*J* suggests the dominance of hole consumption in trap-assisted recombination (Fig. 4b). The electron injection of wide-*E*_g_ QDs is accelerated by the Coulombic attraction of the hole in a 1S_h_ state at a higher electrical bias, where the slope in EL intensity–*J* curve changes from 2.5 to 1. On the other hand, in the case of TCTA-based QLEDs, the injection of both carriers takes place under sufficient bias (*V*_L, TCTA_ = 1.9 V). At *V* ≥ *V*_L_, electrons introduced in overall QDs attract the holes and vice versa, enabling all QDs to contribute to the EL and yielding the linearity in the EL intensity–*J*, as shown in Fig. 4c. Low hole concentration at the QD–TCTA junction leads to fewer trap-involved recombination events.

In this sense, our interpretation addresses the discrepancy raised in the continuum DOS model for 2TNATA-based devices. The narrow-*E*_g_ QDs give off EL as the correlated, rapid injection of a hole outpaces the trap-assisted recombination, while the EL from wide-*E*_g_ QDs is suppressed due to the trap recombination pathway. As a result, the EL spectrum is skewed to exhibit disproportionately high emission intensity at lower end of the energy even at an elevated bias.

Our findings provide a window with which to view the operation of hybrid QLEDs from both macroscopic and microscopic perspectives. Of particular interest is the condition in which barrierless, balanced carrier injection can take place. From the macroscopic viewpoint, the core/shell QDs act as a class of semiconductors and modify the band structure of surrounding CTLs along the surface state-pinned E_F_. The resulting electrostatic potential gain in the hybrid QLEDs is the main driving force for barrierless hole injection. From the microscopic perspective, the charge injected into individual QDs provides marginal local electrostatic potential gain for the opposite charge via Coulombic interaction. When only the injection barrier exceeds the extent of Coulombic attraction, the discrete nature of QDs becomes significant, only to cause uneven carrier injection, hence poor EL performance of QLEDs (as demonstrated in the 2TNATA device; see Supplementary Fig. 8). For the efficient operation of QLEDs, therefore, the injection barrier should be kept lower than the extent of Coulombic interaction. For example, our explanation offers hindsight as to recent high-performance InP QLEDs^3^ using ZnMgO ETLs with reduced electron affinity. We envision rigorous and thorough study on surface modification, e.g., via ligand exchange, as a definite must in the way of taking the efficiency and stability of QLEDs up a notch.

## Methods

### Materials

Indium acetate (In(Ac)_3_, 99.99%), zinc acetate (Zn(Ac)_2_, 99.99%), tris(trimethylsilyl)-phosphine ((TMS)_3_P, 99.9%), 1-octadecene (ODE, 99%), oleic acid (OA, 99%), and tri-n-octylphosphine (TOP, 99%) were purchased from UniAm. Selenium (Se, 99.99%), sulphur (S, 99.98%), zinc acetate dihydrate (Zn(Ac)·2H_2_O, 99%), potassium hydroxide (KOH, 99.99%), 1-octanethiol (OT, 98.5%), zinc chloride (ZnCl_2_, 99.995%), oleylamine (OLA, 70%), trioctylphosphine oxide (TOPO, 99%), ethanolamine (EA, 99%), and lithium triethylborohydride (LBH, 1 M in THF) were purchased from Sigma Aldrich. All anhydrous organic solvents were purchased from Daejung (Korea) and used after N_2_ purging. Organic hole injection layers were purchased from Lumtec. Molybdenum trioxide (MoO_3_, 99.999%) and Al (99.999%) were purchased from Alfa Aesar. Au (99.99%) was purchased from iTASCO. Unless noted otherwise, chemicals were used as received without further purification.

### Synthesis of InP/ZnSe/ZnS QDs

All precursor solutions were prepared under inert atmosphere. We prepared 0.5 M In(OA)_3_ by degassing a mixture of 20 mmol of In(Ac)_3_ and 19.7 mL of OA in a round-bottom flask at 130 °C for 3 h and subsequently diluting the mixture with 20.3 mL of ODE. We prepared 0.5 M Zn(OA)_2_ by reacting 20 mmol of Zn(Ac)_2_ with 13 mL of OA at 120 °C under vacuum for 3 h and adding 27 mL of ODE. To prepare 2 M TOPSe (or 2 M TOPS), we mixed 20 mmol of Se (or S) with 10 mL of TOP, and then heated the mixture at 180 °C for 2 hrs. All precursor solutions were stored in a nitrogen-filled glove box prior to use.

We synthesized InP cores by following a previously reported recipe^15^. In a three-neck round bottom flask, 20 mL of 0.5 M In(OA)_3_ and 150 mL of ODE were loaded and degassed at 110 °C for 2 h. After the reaction vessel was filled with N_2_, we injected a mixture of 10 mL of 0.5 M (TMS)_3_P in TOP into the reaction flask, which was then heated up to 260 °C and kept for 10 min. To obtain InP cores with a mean diameter of 3.4 nm, a mixture of 15 mL of 0.5 M In(OA)_3_ and 15 mL of 0.5 M (TMS)_3_P was slowly added until the first exciton peak reaches ca. 570 nm. After quenching the reaction mixture, we purified the crude solution twice by the precipitation (acetone)/redispersion (toluene) protocol. The resultant InP cores were dissolved in hexane (concentration: 100 mg mL^−1^) and stored in a glove box for further use.

For the ZnSe/ZnS shell growth, 4 mL of 0.5 M Zn(OA)_2_ and 10 mL of ODE were degassed at 110 °C for 2 h. After back-filling with N_2_, 0.5 mL of as-prepared InP core dispersion was injected to the flask at 180 °C and aged for 30 min. At 300 °C, 1.0 mL of TOPSe was introduced dropwisely and kept for an hour to grow 2 nm-thick ZnSe shell. To complete with 1 nm-thick ZnS shell, 8 mL of 0.5 M Zn(OA)_2_ and 2 mL of 2 M TOPS were added to the reactor and waited for an hour. After the reaction is terminated, we purified the crude solution once using acetone and toluene. And added 0.1 mL of OT to improve colloidal stability during extensive purification. After purifying OT-treated QDs four times, the resulting QD dispersion in n-octane was stored in a glove box for device fabrication.

### Synthesis of ZnS NCs

We synthesized ZnS NCs via a previously reported synthetic protocol with slight modification^42^. Briefly, 8 mmol of ZnCl_2_, 40 mL of OLA, and 9.2 g of TOPO were degassed at 150 °C for 2 h. Separately, 36 mmol of S and 15 mL of OLA were reacted at 150 °C for 1 h under vacuum. After quenching precursor solutions, 2.5 mL of S precursor solution was added to the Zn precursor solution and was heated to 320 °C. After an hour, the crude solution was quenched to room temperature and purified using acetone and toluene twice. To coincide the surface chemistry of ZnS NCs’ with that of QDs, as-prepared QDs were treated with Zn(OA)_2_ and TOPS, precursors used for the outmost ZnS shell. The overall ZnS NCs were mixed with 8 mL of 0.5 M Zn(OA)_2_ and 40 mL of ODE and degassed at 120 °C for 2 h. After back-filling with N_2_, 2 mL of 2 M TOPS was injected into the solution at 300 °C and rested for an hour, which yields a mean diameter of 8.4 nm. We purified the final product once with acetone and toluene, and treated with OT, as described above. The purified ZnS NCs were dissolved in *n*-octane and stored in the glove box.

### Synthesis of ZnO NPs

We prepared ZnO NPs using a recipe published by Kwak et al.^6^ with minor modification. 2.5 g of Zn(Ac)_2_·2H_2_O was dissolved in 100 mL of methanol at 60 °C for 30 min. 1.25 g of KOH dissolved in 50 mL of methanol was separately prepared and added to the Zn(Ac)_2_·2H_2_O solution at 60 °C. After 150 min, white precipitate was collected by centrifugation and washed with methanol twice. To improve colloidal stability, the product was treated with 7 mL of ethanolamine solution (10 vol% in methanol) for 5 min. To remove excess ethanolamine and adjust the concentration of the dispersion of ZnO NPs, we precipitated the product using a mixture of methanol, hexane, and isopropanol (=1:6:1 in volume ratio) and redispersed in desired amount of ethanol.

### Characterization of InP/ZnSe/ZnS QDs and ZnS NCs

We obtained TEM images using an FEI Tecnai F20 electron microscope. Absorption and PL spectra were acquired using a Shimadzu UV-3600 spectrometer and a HORIBA Fluoromax-4 spectrometer. We measured absolute PL QY using a Hamamatsu, Quantaurus-QY C11347 spectrometer equipped with an integrating sphere, by exciting the samples at the wavelength of 450 nm. PL QY of QDs is 72% in solution and 41% in film on the glass.

### Fabrication of QLEDs

Indium–tin–oxide (ITO) substrates were washed with DI water, acetone, IPA and dried in a convection oven at 120 °C. ZnO ETL was deposited by spinning 20 mg mL^−1^ of ZnO solution on top of an ITO substrate at 4000 rpm for 30 s and dried at 180 °C for 30 min, to yield a layer of about 30 nm thickness. QD EMLs with a thickness of 18 nm was deposited by spun the QD dispersion on the ITO/ZnO substrates in the same way above. We then transferred ITO/ZnO/QD substrates to a thermal evaporator, in which, organic HTLs (60 nm), MoO_*x*_ (10 nm), and Al (100 nm) were sequentially deposited at 1 × 10^6^ Torr on the substrates with an evaporation rate of 1.0, 0.2–0.3, and 2.0 Å s^−1^, respectively. The QLEDs were encapsulated with UV-curable resin and kept in a glove box before use. For the ZnS-employed device, the 18 nm of ZnS NC film was deposited likewise, where ZnS NCs simple replace QDs in the EMLs. We notify that the thin film fabrication and sample handling were thoroughly performed in N_2_ atmosphere (O_2_ and H_2_O < 0.1 ppm).

### Device characterization

*J*−*V*−*L* characteristics and power spectral density of EL were collected using a Konica Minolta CS-2000 spectroradiometer coupled with a Keithley 2400 source meter. *V*_J_ is determined by a voltage where *J* is transformed from Ohmic current to Trap-filled current. *V*_L_ is a voltage where the light output from the device exceeds 1 × 10^−3^ cd m^−^^2^, the detection limit of the spectroradiometer. Spectrally resolved EL intensity can be directly acquired from the power spectral density of EL at 1.7, 2.0, and 2.2 eV for each data point. The capacitance was measured using an Ivium Technologies Vertex potentiostat by sweeping DC voltage with sinusoidal AC frequency of 100 Hz and amplitude of 0.1 V.

### Time-resolved PL analysis

Decay dynamics of global PL spectra were obtained using a Picoquant time-correlated single-photon counting system (TCSPC) which employed a pulsed laser diode at 488 nm and single-photon avalanche diodes acquiring photons from 500 nm to 1100 nm. The excitation pulse had a resolution of 200 ps and a repetition rate of 500 kHz. This low-energy excitation allowed for probing decay dynamics of QDs in ITO/ZnO/QD and ITO/ZnO/QD/HTLs samples without the participation of carriers generated in HTLs (Fig. 2c, f).

The spectrally-resolved PL decay of ITO/ZnO/QDs (Fig. 4a) was performed using a Horiba DeltaTime TCSPC kit, where the samples were excited at 402 nm using a laser diode with a resolution of 200 ps and a repetition rate of 1 MHz. At the emission slit, signals were collimated by a monochromator with a bandpass of 3 nm and detected by a photomultiplier tube.

Negative trion decay of InP/ZnSe/ZnS QDs was characterized using LBH as an electrochemical dopant^23^. Briefly, in presence of LBH in the QD dispersion, ultraviolet light at 365 nm was illuminated for several minutes to cause photo-doping. To prevent the formation of doubly charged QDs, average electron occupancy was limited to less than 0.3. A PL decay trace of neutralized QDs was acquired by exposing QD dispersion to air. By subtracting tail-normalized PL decay traces of charged and neutral QDs, we obtained negative trion decay dynamics with $\tau_{{{{\mathrm{X}}}}^{-}}$ = 0.9 ns, as shown in Supplementary Fig. 5.

PL decay lifetimes and normalized pre-exponential factors for X, X^−^, and ET are quantified using a tri-exponential decay model:

$$ y=\mathop{\sum} _{{i=}\atop{{{{{\rm{X}}}}}},{{{{{\rm{X}}}}}}^{-}\,{{{{{\rm{and}}}}}}\,{{{{{\rm{ET}}}}}}}{A}_{j}{e}^{-t/{\tau }_{i}}$$

where *A*_i_ is a pre-exponential factor of *i* and is relevant to the contribution of *i* to the entire decay process, τ_i_ is a recombination lifetime of *i*, *t* is time, and *i* is a decay channel of X, X^–^, and ET. For convenience, we normalized the amplitude of PL intensity to 1 to establish ∑*A*_i_ = 1. Assuming that *τ*_X_ and $\tau_{{{{\mathrm{X}}}}^{-}}$ of QD films are identical to those of QD in solution and *τ*_X_ = 29 ns and $\tau_{{{{\mathrm{X}}}}^{-}}$ = 0.9 ns, we obtained *τ*_ET_, *A*_X_, $A_{{{{\mathrm{X}}}}^{-}}$, and *A*_ET_ with high credibility of fit (See Supplementary Table 2). ${{{\mathrm{A}}}}_{{{{\mathrm{X}}}}^{-}}$ can be considered as *f*_e_.

### Ultraviolet photoelectron spectroscopy

ITO substrate was prepared by the washing protocol for QLEDs. A 150 nm-thick Au-coated Si substrate was prepared by thermal evaporation of Au at 1 × 10^−^^6^ Torr with an evaporation rate of 1.0 Å s^−1^. ZnO, QDs, and ZnS NCs thin films were deposited on the substrates by spinning each dispersion at 4,000 rpm for 30 s in an N_2_-filled glove box. To investigate thickness-dependent energy level modification of HTLs, we thermally evaporated 5, 10, and 20 nm-thick HTL thin films on top of ITO/ZnO/QD substrates with an evaporation rate of 1.0 Å s^−1^. To avoid surface contamination or degradation, the entire preparation and handling off all samples took place in N_2_ atmosphere (O_2_ and H_2_O < 0.1 ppm) until the sample was directly loaded to the ultra-high vacuum chamber for the UPS analysis. The UPS spectra were taken using Thermo VG Scientific Sigma Probe with He I light source (*hv* = 21.2 eV). The Fermi levels of samples were calibrated with respect to that of Au reference. We determined the cutoff energy (*E*_cutoff_) by taking linear extrapolation at the high binding energy region, where the work function = 21.2 − *E*_cutoff_. *E*_F_ − *E*_VBM_ was estimated using the onset energy (*E*_onset_) at low binding energy region and the ionization energy was computed from (*E*_He I_ − E_cutoff_) + *E*_onset_. *E*_VBM_ − *E*_g_ yields *E*_CBM_.

## Supplementary information

Supplementary Information

## Data Availability

The data that support the findings of this study are available from the corresponding author upon reasonable request.
