# Supplementary material for: Surface state-induced barrierless carrier injection in quantum dot electroluminescent devices
Source: Nat Commun. 2021 Sep 27;12:5669. doi: 10.1038/s41467-021-25955-z (PMC8476532; doi:10.1038/s41467-021-25955-z)
Supplement: Supplementary file 1 — Supplementary Information [file 41467_2021_25955_MOESM1_ESM.pdf]

Supplementary Information

Surface State-Induced Barrierless Carrier Injection in Quantum Dot Electroluminescent Devices

Hyeonjun Lee[1], Byeong Guk Jeong[2], Wan Ki Bae[2], Doh C. Lee*[,1], and Jaehoon Lim*[,3]

[1]Department of Chemical and Biomolecular Engineering, KAIST Institute for the Nanocentury, Korea Advanced Institute of Science and Technology (KAIST), Daejeon 34141, Republic of Korea.

[2]SKKU Advanced Institute of Nanotechnology (SAINT), Sungkyunkwan University (SKKU), Suwon, Gyeonggi-do 16419, Republic of Korea.

[3]Department of Energy Science, Center for Artificial Atoms, Sungkyunkwan University (SKKU), Suwon, Gyeonggi-do 16419, Republic of Korea.

*E-mail correspondence to D. C. Lee (dclee@kaist.edu) and J. Lim (j.lim@skku.edu)

Supplementary Note 1. Assignment of electronic states in core/shell QDs.

The penetration depth of UV probe being ~2.5 nm makes the ultraviolet photoelectron spectroscopy (UPS) highly sensitive to surface compositions. Therefore, it is reasonable to presume that photoelectrons from the InP/ZnSe/ZnS QDs used in this study result mainly from the outer-most ZnS shell, as discussed in the main text. However, inhomogeneity of ZnS shell thickness (Supplementary Fig. 1) allows us to detect the energy level position of InP cores. An inset in Supplementary Fig. 2c displays distinctive parasitic signal near the photoelectron emission onset of ZnS shell. The onset taken from this parasitic signal records 1.5 eV that stands for a difference between Fermi level and $1S_h$ state, $E_F - E_{1S_h}$. This value is in agreement with a separate assignment of $1S_h$ from the valence band maximum (VBM) of ZnS[S1] that accounts for the difference of VBM between ZnS and InP, $|E_{VBM, ZnS} - E_{VBM, InP}|$, of 1.15 eV and the hole confinement energy of ca. 0.05 eV: $|E_F - E_{1S_h}| + |E_{VBM, ZnS} - E_{1S_h}| = 1.5$ eV + 1.1 eV = 2.6 eV, identical to the $|E_F - E_{VBM, ZnS}|$. Based on the $1S_h$ position, we can define the $1S_e$ position as 3.98 eV using an optical band gap of QDs, 1.97 eV (Supplementary Fig. 2e).

Supplementary Note 2. Calculation of electrostatic potential granted by partially charged QD emissive layers.

Modified Helmholtz equation[S2] expresses the vacuum level shift by the dipoles between ZnO and charged QDs (Δ_e) in the emissive layer as below:

$$\Delta_e = \frac{d}{\varepsilon_0 \varepsilon_f} \times f_e \times \rho_{QD}, \qquad \text{Equation (S1)}$$

where d is a distance from the center of charged QD to the ZnO-QD interface, ε_0 is a vacuum permittivity, ε_f is a dielectric constant of closed-packed QD film, f_e is a fraction of negatively charged QDs, and ρ_{QD} is an area density of QD emissive layer.

Assuming that the two-monolayer-thick QD emissive layer is composed of hexagonal closed-packed and charged QDs are randomly distributed, ρ_{QD} is 2.85×10^{16} m^{-2} and f_e is 0.15 for each QD layer (Fig. 2c). d is 5.7 nm for bottom QD layer and 15.7 nm for top QD layer including surface ligand length of 1 nm.

We adopt the Bruggeman effective medium model[S3] to approximate ε_f. This model is known to calculate dielectric constant for thin film with high volume fraction of QD.[S4] ε_f can be calculated as follows:

$$\varepsilon_f = (\beta + \sqrt{\beta^2 + 8\varepsilon_{QD}\varepsilon_{mat}})/4 \qquad \text{Equation (S2)}$$

$$\beta = (3\eta_{QD} - 1)\varepsilon_{QD} + (3\eta_{mat} - 1)\varepsilon_{mat} \qquad \text{Equation (S3)}$$

$$\eta_{QD} = A \times \frac{4/3\pi r^3}{4/3\pi(s/2)^3} \qquad \text{Equation (S4)}$$

ε_{QD} is 10 for an average value of static dielectric constants for bulk InP, ZnSe, and ZnS. ε_{mat} is 2 for dielectric constant of matrix composed of air and organic ligand. η_{QD} is volume fraction of QDs in an emissive layer and η_{mat} is volume fraction of matrix, $\eta_{mat} = 1 - \eta_{QD}$. *A* is a packing fraction of hexagonal close-packed QDs, *r* is an average radius of QDs and *s* is a QD-to-QD spacing. The resultant ε_f is 4.3 and the overall Δ_e yields 0.18 eV by summing Δ_es for each QD layer.

Supplementary Note 3. Computation of electroluminescence spectra of QLEDs.

To simulate electroluminescence spectra as a function of applied bias, we assume that i) the $1S_e$ states of QDs in the emissive layer are largely varied from dot to dot while those of holes are identical because of a large difference of electron and hole effective masses (m_h^*/m_e^* = ~7.5), ii) PL spectrum of QD emissive layer reflects the $1S_e$ density of states, iii) electron injection rate is a bottleneck to generate excitons anticipated from energy level landscape in Fig. 3, iv) the neutral excitons are solely generated during device operation, and v) all QDs occupied by an exciton equally contribute to EL spectra. Those assumptions allow us to regard that the EL spectrum directly reflects the $1S_e$ density of states occupied by electrons.

Electron-filled $1S_e$ states, $\sigma(E, \mu, T)$, can be expressed as below:

$$\sigma(E, \mu, T) = \rho_e(E) \times F(E, \mu, T), \quad \text{Equation (S5)}$$

where E is energy, μ is an electrochemical potential, T is temperature, $\rho_e(E)$ is a $1S_e$ density of states in a QD emissive layer and $F(E, \mu, T)$ is the Fermi-Dirac distribution function. Here, ρ_e can be taken from PL spectrum of QD emissive layer (Supplementary Fig. 1a).

Since the surface states are randomized from dots to dots, the Fermi levels of each QD should be distributed, not a single value. To weight the different surface state of each QDs to overall $1S_e$ density of states, we employ the defect distribution function $\delta_{surface}(E_c)$ that contains the fraction of QDs with a Fermi level position at E_c. Assuming that the surface state emission of ZnS NCs is $\delta_{surface}(E_c)$ (Supplementary Fig. 2a), the apparent $1S_e$ states ($\rho_{e,\, app}$) and electron occupation, $\sigma_{app}(E, \mu, T)$, is deduced as below:

$$\rho_{e,\, app}(E) = \int \rho_e(E, E_c)\, \delta_{surface}(E_c)\, dE_c. \quad \text{Equation (S6)}$$

$$\sigma_{app}(E, \mu, T) = \rho_{e,\, app}(E) \times F(E, \mu, T). \quad \text{Equation (S7)}$$

Supplementary Note 4. Calculation of polarization and Coulomb repulsion energy.

To consider the energy level modification induced by carrier occupation in InP cores, we calculate the polarization energy (Σ) and the Coulomb interaction energy (U) by ref S5 with an assumption that charges are fully confined in the core.

Σ granted by a single carrier present in QD can be written as below:

$$\Sigma(R) = \frac{\varepsilon_{in}-\varepsilon_{out}}{\varepsilon_{in}(\varepsilon_{in}+\varepsilon_{out})}\left(\frac{1}{\alpha} - 0.376\alpha + 0.933\right)\frac{e^2}{8\pi\varepsilon_0 R} \quad \text{Equation (S8)}$$

$$\alpha = \frac{\varepsilon_{out}}{\varepsilon_{in}+\varepsilon_{out}} \quad \text{Equation (S9)}$$

ε_{in} and ε_{out} are bulk dielectric constants of confined nanocrystal (dielectric constant for InP = 12.5) and embedding matrix (dielectric constant for QD film = 4.3; see the Supplementary Note. 2), respectively. The e is a unit charge and ε_0 is a vacuum permittivity. R is an average radius of InP cores, 1.75 nm. Σ is calculated to be merely 28 meV, so the polarization barely hinders the initial carrier injection process to QDs.

U between a carrier residing in the core and a newly-added carrier can be calculated by below:

$$U(R) = \left(\frac{1}{\varepsilon_{out}} + \frac{0.79}{\varepsilon_{in}}\right)\frac{e^2}{4\pi\varepsilon_0 R} \quad \text{Equation (S10)}$$

The calculated U is as much as 0.244 eV. Thus, as long as one or more carriers are present in a QD, the carrier injection of same polarity becomes difficult due to Coulombic repulsion. On the other hand, the carrier injection of opposite polarity is promoted by Coulombic attraction (–U). For the QD with 2.2 eV E_g (*i.e.*, wide-E_g QDs in QD ensemble used in our study), the effective electron injection barrier in 2TNATA device is reduced from ~0.5 eV to ~0.26 eV when a hole is introduced. The effective hole injection barrier is decreased from ~0.1 eV to ~–0.14 eV by the pre-existing electron.

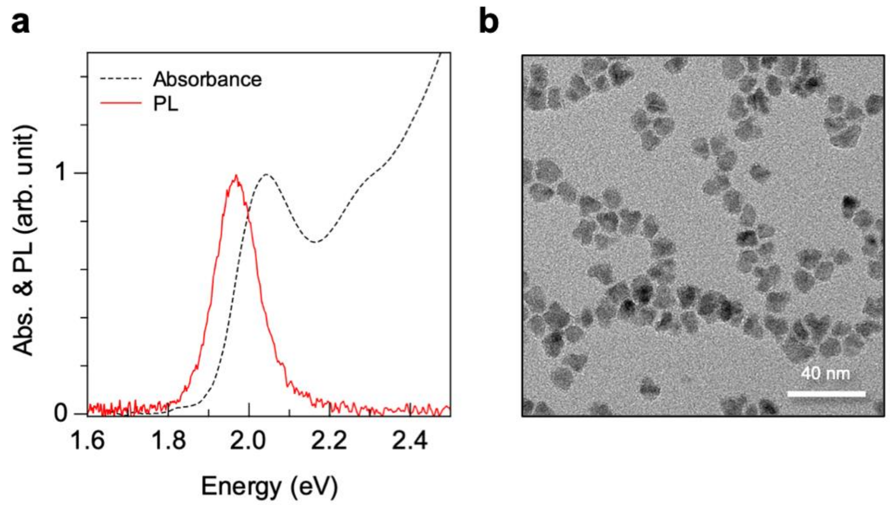

Supplementary Fig. 1| Characteristics of InP/ZnSe/ZnS quantum dots used in this study. **a**, Absorption spectrum (black dashed line) and photoluminescence spectrum (PL, red solid line) in which PL is centred at 627 nm (1.97 eV) with a full width at half maximum of 40 nm (0.11 eV). InP core diameter is 3.4 ± 0.3 nm and overall diameter of core/shell QDs is 9.4 ± 1.7 nm. **b**, Transmission electron microscopy image of QDs.

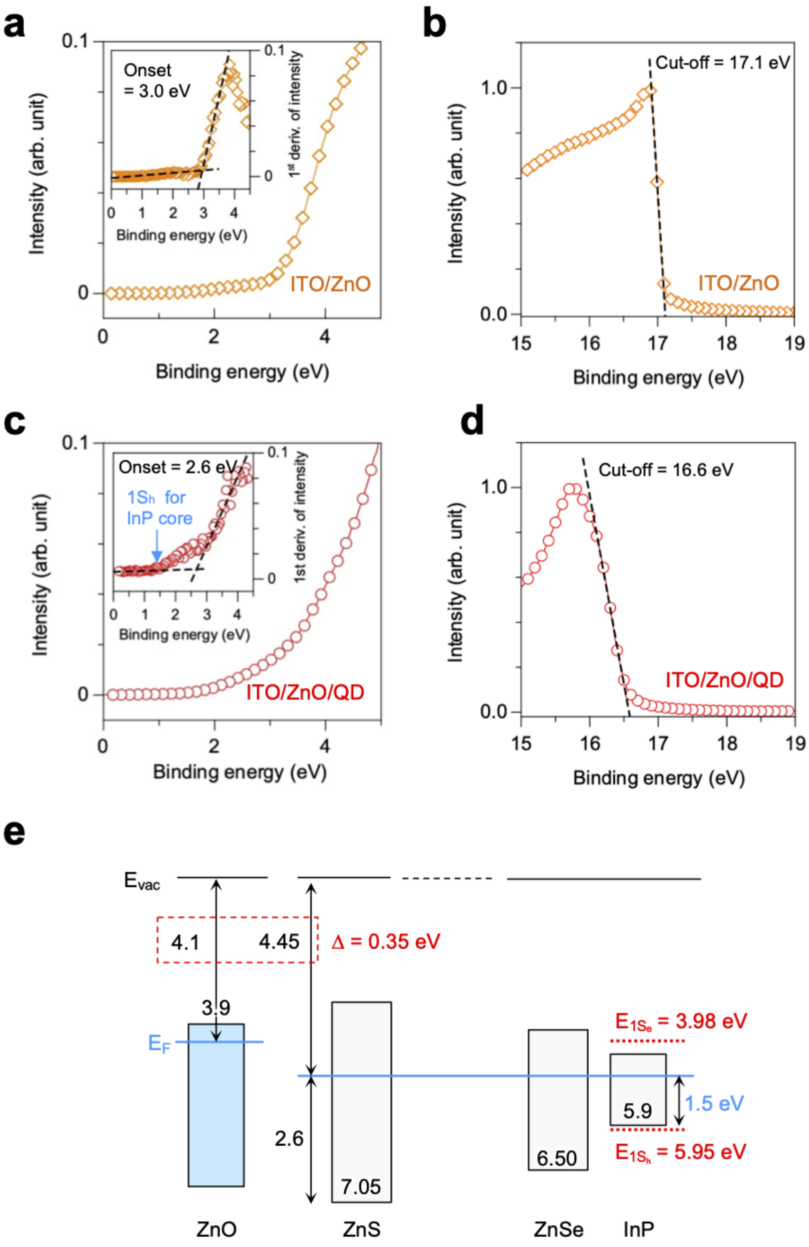

Supplementary Fig. 2| Determination of Energy Level Landscape at ZnO/QD interface using ultraviolet photoelectron spectroscopy. Photoelectron spectra of ITO/ZnO (**a** and **b**) and ITO/ZnO/QDs substrates at low binding energy (**a** and **c**) and high binding energy cut-off (**b** and **d**) regime. Inset in **a** and **c** is a 1st derivative of photoelectron intensity to clarify the onset.[S6] Satellite signal at ca. 1.5 eV in the inset of **c** originates from contribution of InP cores. **e**, Assignment of band position and Fermi level (E_F) of ZnO ETL, ZnS, ZnSe and InP with respect to vacuum level (E_{vac}). The bulk energy levels of InP, ZnSe, and ZnS (black lines) are referred to ref. S1. The difference of Fermi levels between ZnO and ZnS without junction formation is 0.35 eV. See Supplementary Note 1 for assignment of $1S_e$ and $1S_h$ states.

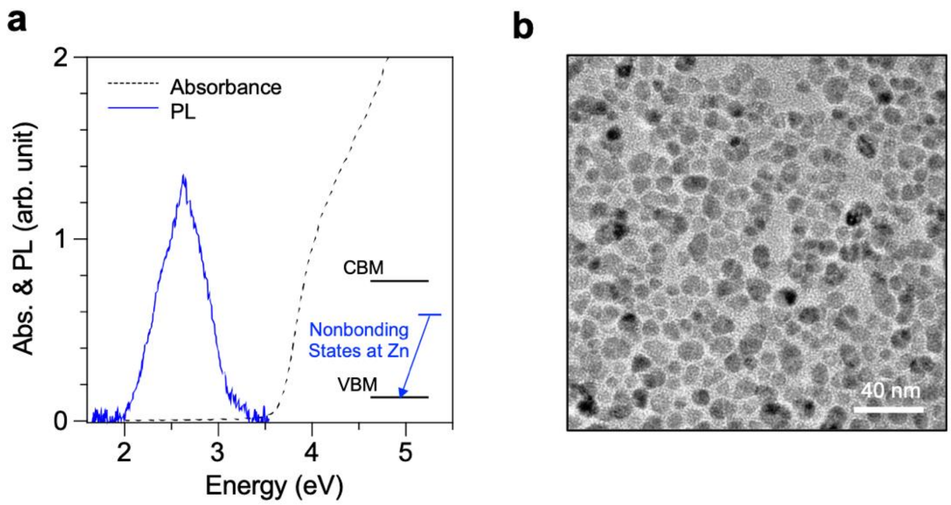

Supplementary Fig. 3| Characteristics of ZnS nanocrystals. a, Absorption (black dashed line) and photoluminescence (PL, blue solid line) spectra of ZnS nanocrystals which PL centred at 2.62 eV with emission bandwidth of 0.51 eV. **b**, Transmission electron microscopy image of ZnS nanocrystals.

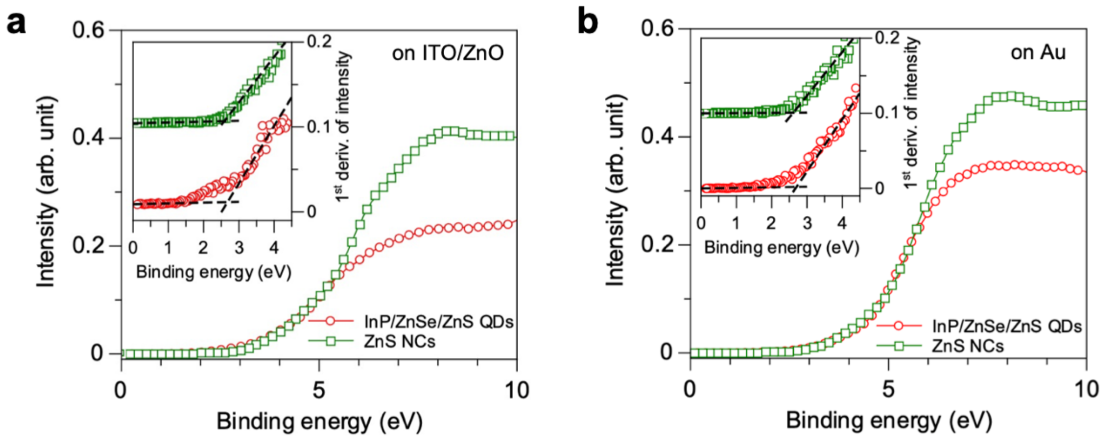

Supplementary Fig. 4| Analysis on valence band maximum position of InP/ZnSe/ZnS QDs and ZnS NCs on different substrates. Photoelectron spectra at low binding energy region of QDs and ZnS NCs on **a**, ITO/ZnO and **b**, Au substrates. Insets show indistinguishable onsets at 2.6 eV between two materials, suggesting they have same positions of valence band maximum from Fermi level.

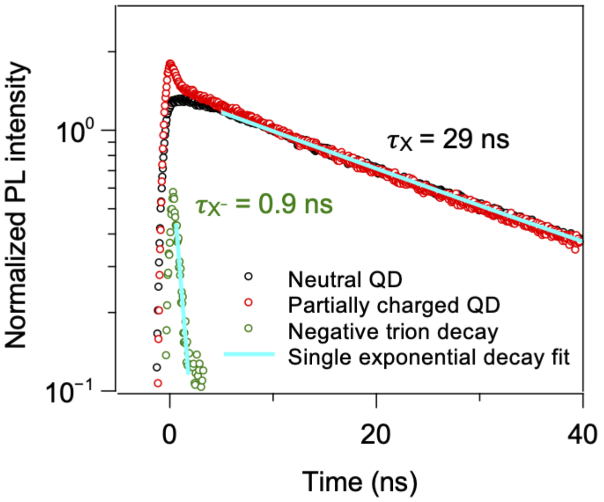

Supplementary Fig. 5| Determination of single exciton and negative trion lifetimes of InP/ZnSe/ZnS QDs. Tail-normalized PL decay traces of neutral (black), charged (red) QDs. Subtraction of two traces extracts the negative trion (X) decay trace (green circles). The τ_X and τ_{X^-} were extracted by single exponential decay fit (sky blue). An average occupancy of extra electrons in QDs was ca. 0.3. All samples were excited using a pulsed laser diode at 402 nm.

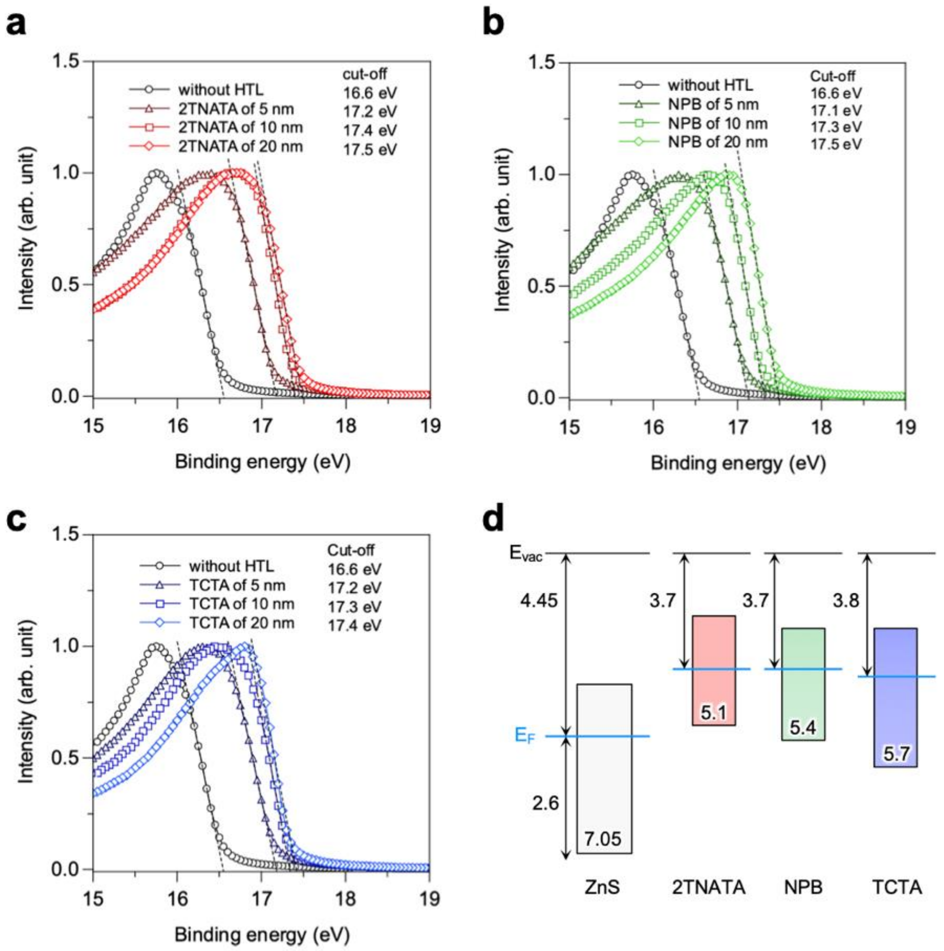

Supplementary Fig. 6| Determination of energy level landscape of various hole transporting layers on quantum dots. Ultraviolet photoelectron spectra at high binding energy region for **a**, 2TNATA, **b**, NPB and **c**, TCTA for thicknesses of 0 nm (QD only, open circle), 5 nm (open triangle), 10 nm (open square), and 20 nm (open diamond). Cut-off values are listed as inset for clarity. **d**, Assignment of band position of various hole transport layers on ITO/ZnO/QDs substrates without junction formation. Work functions of bulk 2TNATA, NPB and TCTA films are based on the UPS spectra for 20 nm thick HTLs.

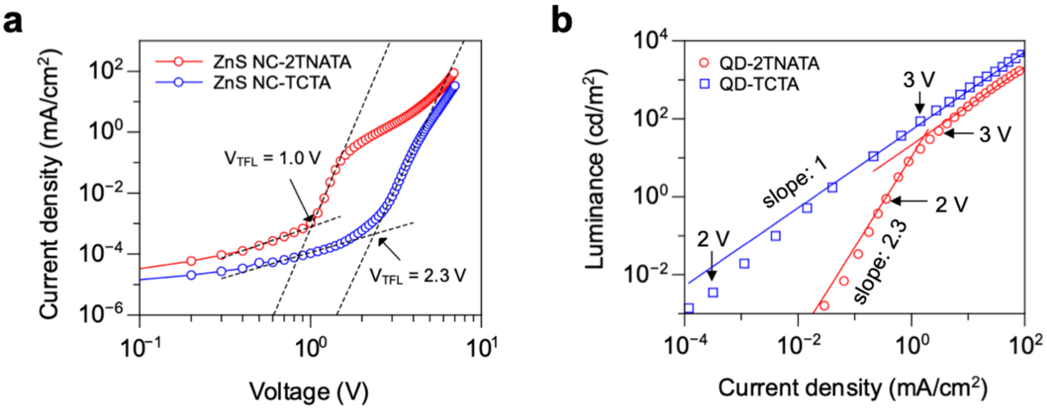

Supplementary Fig. 7| Trap-assisted recombination characteristics of devices. a, *J*–*V* characteristics of ITO/ZnO/ZnS NCs/HTLs/MoO_x/Al, where HTLs are 2TNATA (red) and TCTA (blue). Trap-filled limit voltages (V_{TFL}s) are 1.0 V and 2.3 V for 2TNATA- and TCTA-based devices, respectively. **b**, *L*–*J* characteristics of InP/ZnO/QDs/HTLs/MoOx/Al: red for 2TNATA and blue for TCTA (blue). The super-linear (~2.3) behaviour observed in 2TNATA-based QLEDs implies the involvement of surface trap-derived carrier loss pathways.

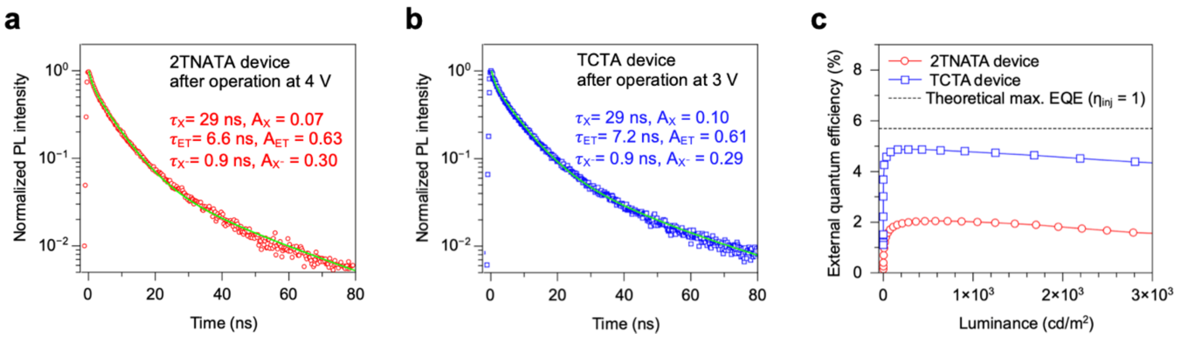

Supplementary Fig. 8| Assessment of device efficiency of QLEDs. PL decay dynamics of QD emissive layers in QLEDs employing **a**, 2TNATA (red) and **b**, TCTA (blue). Decay traces were collected immediately after termination of devices that were operated at their peak efficiency condition for 1 min (3 V for TCTA and 4 V for 2TNATA device). An excitation was 488 nm to avoid involvement of HTL excitation. The fraction of charged QDs (f_e), equivalent to A_{X^-}, were obtained from tri-exponential fitting (green) (See Method for details). **c**, External quantum efficiency (EQE) curves of QLEDs with 2TNATA (red) and TCTA (blue). Theoretical EQE limit is indicated using dashed horizontal line at 5.7 %, computed from assumption of $\eta_{inj} = 1$, $\eta_{out} = 0.2$ and $\eta_{QY, EML} = \eta_{QY, film} \times (1 - f_e) = 0.29$.

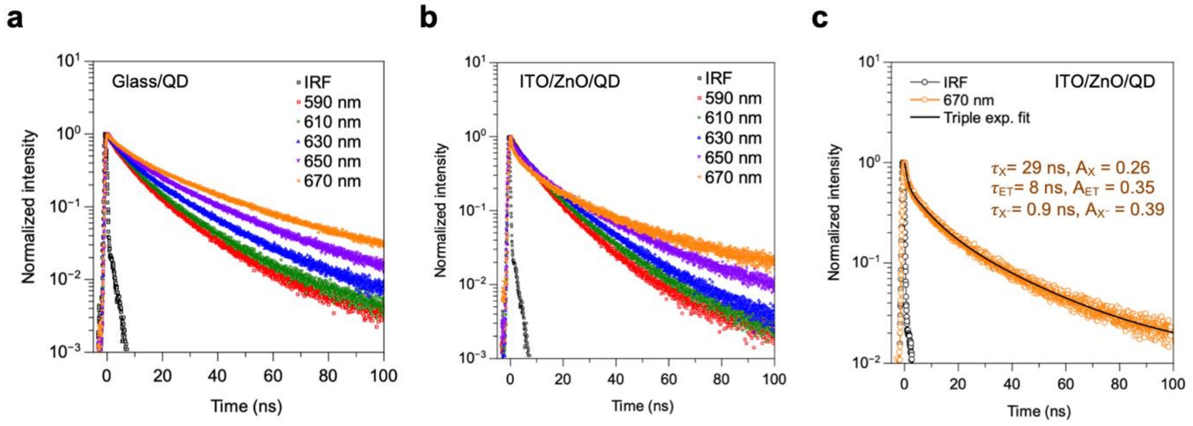

Supplementary Fig. 9| Spectrally resolved PL decay of InP/ZnSe/ZnS QD films on various substrates. PL decay curves acquired on **a**, bare glass and **b**, ITO/ZnO substrate with an excitation of 402 nm. PL decays were probed at different wavelengths: 590 nm (red), 610 nm (green), 630 nm (blue), 650 nm (purple) and 670 nm (orange). **c**, PL decay for ITO/ZnO/QD probed at 670 nm and its tri-exponential fitting result. Instrument response functions (IRFs) are also included for each measurement.

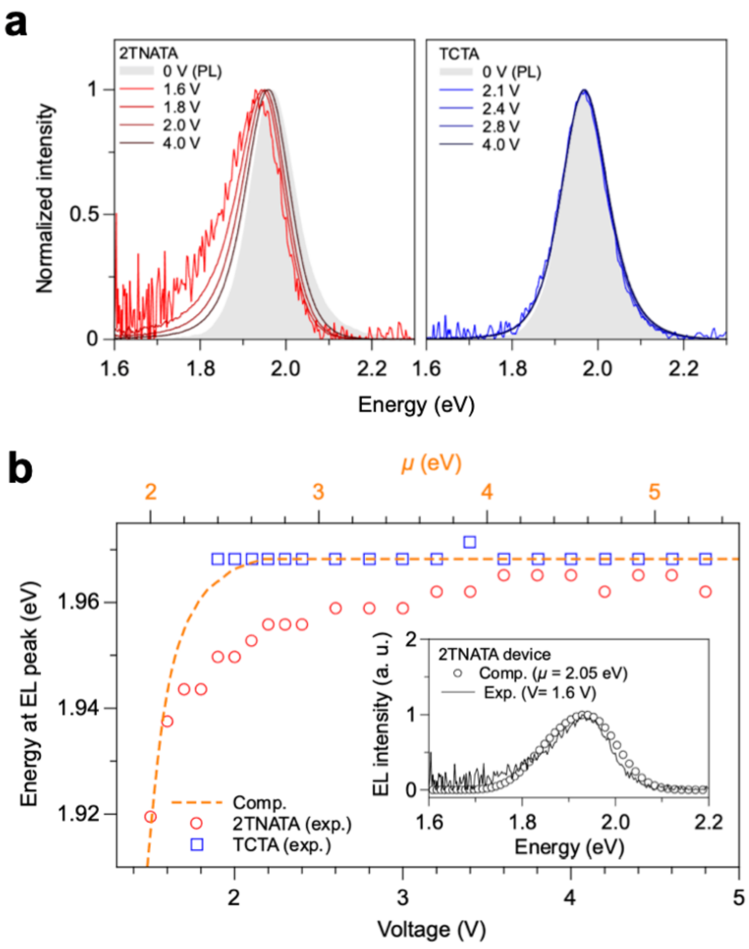

Supplementary Fig. 10| Experimentally observed and simulated EL spectra of QLEDs. a, EL spectra development of QLED with 2TNATA (left) and TCTA (right) acquired at varied biases. PL spectrum of QD film is provided as grey background for comparison. 2TNATA-based device exhibits asymmetric EL shape even at $V \geq 2$ V. **b**, EL peak positions of 2TNATA- (red circle) and TCTA-based QLEDs (blue square) as a function of applied bias. Simulated EL peak positions (orange dashed line) are also included. Inset shows the computed (open circle) and experimental EL spectra (solid line) of QLEDs with 2TNATA at 1.6 V.

Supplementary Table 1| Literature value for operation thresholds of QLEDs

Reference	Class of CTL	Device structure	E_g (eV)	V_J (V)	V_L (V)
Nano Lett. 9, 2532–2536 (2009)	All organic	ITO/spiroTPD/QD/TPBi/Mg:Ag/Ag	1.9	2.4	N/A[a]
			2.0	2.2	N/A[a]
			2.3	2.6	N/A[a]
			2.5	3.1	N/A[a]
			2.7	2.5	N/A[a]
J. Mater. Chem. C 5, 5018–5023 (2017)	All organic	ITO/PEDOT:PSS/TFB/PVK:QD/TPBi/LiF/Al	2.4	4.7	5.5
Nat. Photonics 1, 717–722 (2007)	All organic	ITO/PEDOT:PSS/poly-TPD/QD/Alq3/Ca/Al	2.0	N/A[b]	3.0
			2.1	N/A[b]	3.0
			2.2	N/A[b]	5.0
			2.4	N/A[b]	4.0
Adv. Mater. 21, 1690–1694 (2009)	All organic	ITO/PEDOT:PSS/poly-TPD/QD/TPBi/LiF/Al	2.4	N/A[b]	3.5
Angew. Chemie 118, 5928–5931 (2006)	All organic	ITO/CBP/QD/TAZ/Alq3/Mg:Ag/Ag	2.4	2.6	N/A[a]
Adv. Mater. 19, 3371–3376 (2007)	All organic	ITO/PEDOT:PSS/BiVB-MeTPD/QD/TPBi/CsF/Al	2.1	2.5	3.9
Phys. Rev. B 78, 085434, (2008)	All organic	ITO/TPD/QD/Alq3/Mg:Ag/Ag	2.0	2.7	N/A[a]
Nat. Photonics 9, 259–265 (2015)	hybrid	ITO/PEDOT:PSS/TFB/QD/ZnO/Al	2.3	2.3	2.0
Nat. Photonics 5, 176–182 (2011)	hybrid	ITO/PEDOT:PSS/TFB/QD/TiO_2/Al	2.1	1.5	N/A[b]
Adv. Mater. 32, 2006178 (2020).	hybrid	ITO/PEDOT:PSS/TFB/QD/ZnO/Al	2.0	1.3	1.7
Nat. Photonics 5, 543–548 (2011)	hybrid	ITO/PEDOT:PSS/poly-TPD/QD/ZnO/Al	2.1	1.1	1.6
			2.3	1.1	1.8
			2.6	1.3	2.3
Nat. Photonics 7, 407–412 (2013)	hybrid	ITO/ZnO/QD/NPB/HIL/Al	2.0	1.5	N/A[b]
ACS Nano 13, 8229–8236 (2019)	hybrid	ITO/PEDOT:PSS/TFB/QD/ZnO/Al	2.0	1.8	1.6
Nat. Commun. 10, 765 (2019)	hybrid	ITO/PEDOT:PSS/TFB/QD/ZnO/Al	2.0	1.7	1.6
			2.7	2.3	2.1
Nat. Commun. 11, 1646 (2020)	hybrid	ITO/HIL/TFB/QD/ZnO/Al	1.9	N/A[b]	1.4
Chem. Mater. 31, 3476–3484 (2019)	hybrid	ITO/ZnO/PFN/QD/TCTA/MoO_x/Al	2.0	1.5	1.7
			2.4	2.0	2.0
Nano Lett. 18, 6645–6653 (2018)	hybrid	ITO/PVP:ZnO/QD/TCTA/MoO_x/Al	2.0	1.9	1.8
ACS Nano 13, 11433–11442 (2019)	hybrid	ITO/PEDOT:PSS/TFB/QD/ZnMgO/Al	2.3	N/A[b]	2.0

[a] Not contained data.

[b] Threshold voltages cannot be resolved owing to the linear scaled plot.

Supplementary Table 2| Recombination characteristics acquired from time-resolved photoluminescence decay of each film, where τ_i is a recombination lifetime in nanosecond, A_i is a normalized pre-exponential factor ($\sum A_i = 1$), and *i* is a decay channel such as negative trion (X^-), energy transfer (ET) and neutral exciton (X). Negative trion and neutral exciton lifetimes are fixed using experimental values

	Decay constants (τ_i / A_i)			Goodness of fit
Samples	X^-	ET	X	R^2
Glass/QD	- / -	10 / 0.64	29 / 0.36	0.9989
ITO/ZnO/QD	0.9 / 0.29	8.9 / 0.57	29 / 0.14	0.9992
ITO/ZnO/QD/2TNATA	0.9 / 0.26	8.2 / 0.63	29 / 0.11	0.9990
ITO/ZnO/QD/NPB	0.9 / 0.22	8.8 / 0.66	29 / 0.12	0.9992
ITO/ZnO/QD/TCTA	0.9 / 0.24	8.8 / 0.62	29 / 0.14	0.9992

References

S1. Walukiewicz, W. Intrinsic limitations to the doping of wide-gap semiconductors. *Phys. B Condens. Matter* **302**, 123–134 (2001).

S2. Amsalem, P. *et al.* Role of charge transfer, dipole-dipole interactions, and electrostatics in Fermi-level pinning at a molecular heterojunction on a metal surface. *Phys. Rev. B* **87**, 035440 (2013).

S3. Choy, T. C. *Effective Medium Theory: Principles and Applications*. (Oxford University Press, 2015).

S4. Grinolds, D. D. W., Brown, P. R., Harris, D. K., Bulovic, V. & Bawendi, M. G. Quantum-dot size and thin-film dielectric constant: Precision measurement and disparity with simple models. *Nano Lett.* **15**, 21–26 (2015).

S5. Jdira, L., Liljeroth, P., Stoffels, E., Vanmaekelbergh, D. & Speller, S. Size-dependent single-particle energy levels and interparticle Coulomb interactions in CdSe quantum dots measured by scanning tunneling spectroscopy. *Phys. Rev. B* **73**, 115305 (2006).

S6. Wood, V. *et al.* Selection of metal oxide charge transport layers for colloidal quantum dot LEDs. *ACS Nano* **3**, 3581–3586 (2009).
